# Recruitment and Retention of Mental Health Workers in Ghana

**DOI:** 10.1371/journal.pone.0057940

**Published:** 2013-02-28

**Authors:** Helen Jack, Maureen Canavan, Angela Ofori-Atta, Lauren Taylor, Elizabeth Bradley

**Affiliations:** 1 School of Public Health, Yale University, New Haven, Connecticut, United States of America; 2 University of Ghana Medical School, Accra, Ghana; Federal University of Rio de Janeiro, Brazil

## Abstract

**Introduction:**

The lack of trained mental health workers is a primary contributor to the mental health treatment gap worldwide. Despite the great need to recruit and retain mental health workers in low-income countries, little is known about how these workers perceive their jobs and what drives them to work in mental health care. Using qualitative interviews, we aimed to explore factors motivating mental health workers in order to inform interventions to increase recruitment and retention.

**Methods:**

We conducted 28 in-depth, open-ended interviews with staff in Ghana’s three public psychiatric hospitals. We used the snowballing method to recruit participants and the constant comparative method for qualitative data analysis, with multiple members of the research team participating in data coding to enhance the validity and reliability of the analysis. The use of qualitative methods allowed us to understand the range and depth of motivating and demotivating factors.

**Results:**

Respondents described many factors that influenced their choice to enter and remain in mental health care. Motivating factors included 1) desire to help patients who are vulnerable and in need, 2) positive day-to-day interactions with patients, 3) intellectual or academic interest in psychiatry or behavior, and 4) good relationships with colleagues. Demotivating factors included 1) lack of resources at the hospital, 2) a rigid supervisory hierarchy, 3) lack of positive or negative feedback on work performance, and 4) few opportunities for career advancement within mental health.

**Conclusions:**

Because many of the factors are related to relationships, these findings suggest that strengthening the interpersonal and team dynamics may be a critical and relatively low cost way to increase worker motivation. The data also allowed us to highlight key areas for resource allocation to improve both recruitment and retention, including risk pay, adequate tools for patient care, improved hospital work environment, and stigma reduction efforts.

## Introduction

Strengthening the mental health workforce is a global priority. The World Health Organization (WHO) estimates that 1.18 million additional mental health workers are needed to close the mental health treatment gap in low- and middle-income countries [1]. Mental, neurological, and substance use (MNS) disorders account for at least 13% of the global burden of disease [2]. More so than other areas of medicine, mental health care relies on trained workers, rather than technology or tools [3]. The WHO’s Mental Health Gap Action Program (mhGAP) and a number of research priority-setting exercises, including the *Lancet* global mental health group and the Grand Challenges in Global Mental Health Initiative, have identified mental health workforce expansion as a key component for improving mental health worldwide [4], [5]. Despite the calls for increased attention to this issue, little research has focused on how to build the mental health workforce, particularly in Africa.

Although recruitment and retention are fundamental to sustaining a robust mental health workforce, we are aware of no studies that have examined how to effectively recruit and retain mental health workers in low- and middle-income countries. Multiple studies document the extensive migration of mental health workers to high-income countries [6]. The existing literature highlights a number of factors that may be responsible for poor recruitment and retention: an inadequate living or working environment, extreme workloads, poor supervision, and disease risk, while other factors may cause workers to stay: higher pay, opportunities for career development, positive feedback, and family or cultural ties [7], [8], [9], [10], [11]. Looking at the mental health workforce specifically, a number of studies highlight negative attitudes of health professionals toward mental health that could limit entry into mental health care [12], [13], [14], [15], [16]. A recent study indicated that Ghanaian medical students thought psychiatry had little prestige and was less lucrative than other specialties, and the majority felt uncomfortable interacting with patients with mental illness. Similarly, many Nigerian physicians sought distance from patients with mental illness, often believing psychiatric patients might be dangerous and attributing mental health disorders to supernatural forces [12]. Missing from the literature, however, are data from mental health workers who have stayed in a low-income country despite the working conditions and stigma. A better understanding of the experiences of mental health workers in low-income countries can inform the design of effective interventions to enhance recruitment and retention, the cornerstones of building the needed mental health workforce in low-income settings, particularly Africa.

Accordingly, we sought to explore motivating factors for mental health workers in Ghana using qualitative interviews conducted with staff at the country’s three public psychiatric hospitals. We selected a qualitative study because there is little previous research on this question, and because we recognized that issues regarding motivating factors may be nuanced and best understood with the rich detail that in-depth, qualitative interviews afford [17], [18]. Findings may be useful for informing the design of policies and practices to enhance recruitment and retention of mental health workers in low-income countries.

## Methods

### Setting

Mental health care in Ghana is government funded, receiving 0.5% of the overall health budget, or about 0.007% of GDP [19], [20]. Ghana has eleven psychiatrists serving a population of nearly 25 million people [21]. Because of the lack of psychiatrists, the country’s approximately 900 psychiatric nurses perform most direct mental health care.

Mental health care services offered by the government take place primarily at the country’s three psychiatric hospitals, which receive nearly 80% of the total government expenditure on mental health services [22] and where this study was conducted. The hospitals are clustered in the costal area near the capital, Accra, and another large city, Cape Coast. Accra Psychiatric Hospital, the largest and oldest of the three hospitals, is located in the center of the capital, Pantang Hospital sits in a rural area just outside the capital, and Ankaful Hospital is located on the outskirts of Cape Coast, 100 miles west of Accra. Each hospital accommodates between 300 and 1200 patients, but may have fewer beds, and a proportion of patients sleep on the floor [20]. The patient capacity and workforce strength of each hospital in 2007 are detailed in **Table 1**, although prompted by recent changes in mental health legislation, the hospitals have been working on reducing the number of inpatients. In-patient treatment is characterized by use of psychotropic medications and long patient stays, with an average stay of 285 days at Pantang Psychiatric Hospital and 82.2 days at Ankaful Psychiatric Hospital (data is not available for Accra Psychiatric Hospital) [22]. The hospitals lack the resources to substantially invest in psychosocial care or counseling.

**Table 1:** Patient capacity and workforce strength of Ghana’s psychiatric hospitals.

Hospital	In-patient capacity [20]	Outpatients seen in 1 year	Psychiatrists employed	Psychiatric nurses employed
Accra Psychiatric Hospital	1200 (800 beds)	8982	4	265
Ankaful Hospital	500 beds	5559	2	166
Pantang Hospital	500 beds	5652	2	160

All data except inpatient capacity came from unpublished data collected as part of the Mental Health and Poverty Project in 2009 to 2010 (outpatients seen in 1 year) and from the researcher’s (HJ) unpublished data collection on the location of Ghana’s psychiatrists and psychiatric nurses (psychiatrists employed and psychiatric nurses employed).

The remaining 20% of mental health funding supports the 3 psychiatrists and the psychiatric nurses who work in Ghana’s regional hospitals and the community psychiatric nurses, who provide direct care and referrals to patients living away from the psychiatric hospitals and follow-up care to patients who have been discharged from the hospitals. Additionally, 70–80% of Ghanaians go to traditional or spiritual healers when facing a mental health problem [23].

### Study Design and Sample

We conducted a qualitative study using semi-structured interviews with staff (n = 28) of Ghana’s 3 psychiatric hospitals during June and July 2011. The staff interviewed worked at Accra Psychiatric Hospital (n = 11), Pantang Hospital (n = 10), and Ankaful Hospital (n = 7) and included psychiatric nurses, nurses in leadership or administrative roles, physicians, and support clinical staff including pharmacists, social workers/psychologists, and medical or nursing assistants. The job title and gender of respondents are displayed in **Table 2**; area of practice, age, and hospital were not included in the table because the small number of staff in some of the positions would make the respondent’s identity clear to anyone familiar with the hospital if those details were revealed, compromising respondent confidentiality. Clinical staff were from a variety of areas of the hospital, including the children’s ward, outpatient department, electroconvulsive therapy department, vagrants’ ward, and general female and male wards. At each hospital, the head administrator was approached and asked to suggest staff members for interviews who collectively varied across the following criteria: length of time working in mental health, position in the psychiatric hospital, gender, and age. Additional participants were determined using the snowballing, or chain referral, method. At the close of the interview, the interviewer asked if the respondent could suggest other staff members who could describe more about what it is like to work at the hospital. The interviewer did not ask for suggestions of staff who satisfied certain characteristics, but, instead, gave the respondent freedom to recommend anyone else who worked at the hospital and who could provide insights into working there. As recommended by experts, the chain referral method allows researchers to target key informants known only to insiders and gain access to previously unknown or unreachable respondents [24].

**Table 2:** Characteristics of interview respondents.

Position	Gender
Senior staff nurse	Female
Staff nurse	Male
Nurse in leading administrative role	Male
Assistant nurse	Male
Senior staff nurse	Female
Psychiatrist	Male
Psychiatrist	Male
Senior staff nurse	Female
Psychiatrist	Male
Senior staff nurse	Female
Nurse in leading administrative role	Female
Administrator	Male
Staff nurse in administrative role	Male
Nurse in leading administrative role	Female
Staff nurse	Female
Community psychiatric nurse	Male
Psychologist	Male
Senior staff nurse	Female
Pharmacist	Male
Psychiatry resident	Male
Senior staff nurse	Male
Physician’s assistant	Male
Trainee in clinical psychology	Male
Psychiatrist	Male
Staff nurse in administrative role	Male
Nurse assistant	Female
Nurse in leading administrative role	Male
Social worker	Male

Before beginning the interview, the interviewer provided the participant with an information sheet about the study, discussed any questions the participants had, and obtained oral consent prior to starting the audiorecording. Oral consent was used to protect the confidentiality of participants. All research procedures were approved by the Institutional Review Board of the Yale School of Medicine and by the Ghana Health Service Ethical Review Committee.

### Data Collection

Interviews were conducted using a semi-structured interview guide (See **Table 3**) with follow-up prompts to elicit additional detail [18], [25]. Using the guide, the interviewer engaged the participant in a 60 to 90 minute conversation about the participant’s reasons for working at a psychiatric hospital, the content of his or her work, and the culture of interpersonal and intergroup relationships in the work setting. When discussing each of these topics, the interviewer asked participants to describe specific scenarios or examples. The interviewer conducted additional interviews until the point of theoretical saturation, when no new concepts emerge with successive interviews [18], [26], [27]. Interviews were audio-recorded upon permission and transcribed by an independent, professional transcriber. In cases where permission for audio recording was not granted, the interviewer took notes by hand and typed the notes soon after the interview.

**Table 3:** Qualitative interview discussion guide.

1. What is your title and how long have you been in this position?
2. How do you fit into the organization? Who supervises you, and whom do you supervise?
3. What did you do before you came to this position? What brought you to this work?
a. Were there any particular experiences during your training or in previous jobs that led you to working with patients with mental illness? That led you to working at this hospital?
b. What training qualified you for your current job?
c. Did you have any training in ethics during school or on the job? If so, how long was it and what was it like?
4. Could you tell me about your role here at the psychiatric hospital? How do you spend your time when you are at work?
a. What are your daily tasks or jobs?
b. How long are your days? When do you come to and leave work?
5. Are there differences between what you thought you were being hired to do and what you actually do? What are those differences?
6. Tell me about your patients. How do you help them?
a. When do you feel most successful in your daily work?
b. Can you describe a good day to me?
c. What was the most difficult dilemma that you had to face at work?
7. What keeps you working here? Why do you stay in this position?
8. I want to learn more about how the people you work with in the hospital interact with one another. Can you describe to me a little bit about the culture of this place? What does it feel like to work here?
a. Supervision
b. Teamwork
c. Interdepartmental communication
d. Accountability
e. Learning
f. Feedback
g. Positive and negative aspects
h. How could it be improved? What would the ideal work environment be like?
9. Is there anything else you would like to share to help me understand your work?

### Data Analysis

We used the constant comparative method for qualitative data analysis [26] as applied to health services research [28]. First, we inductively developed the code sheet. Three members of the research team (EB, MC, HJ) separately did a line-by-line review of 4 randomly selected transcripts. Whenever a new concept appeared in the text, the reader assigned a code. After individually assigning codes, the 3 readers met to develop a common list of codes through discussion of individual code assignments. After developing the initial code list, two coders (MC, HJ) separately read each transcript and assigned codes to sections of text that represented a concept captured by one of the codes. Coded sections varied in length from a phrase to a multi-paragraph block, depending on how long it took the respondent to articulate a single idea that fit within the code’s concept [26]. The coders held a series of meetings to reconcile their codes, coming to a consensus about each code assignment. While assigning codes, the coders referred back to previously coded transcripts to ensure that code assignments were consistent across all interviews. When a concept not captured by one of the codes became apparent, we added a new code to the code sheet to characterize that idea until the code sheet captured all concepts in the 28 interviews. As codes were updated, the coders re-reviewed each previously coded transcript with the updated coding scheme. We used ATLAS.ti Scientific Software, version 6.1 (ATLAS.ti, Berlin, Germany) to facilitate data retrieval, comparison of concepts within each code, and identification of key quotations.

## Results

### Overview

Motivating and demotivating factors for workers to remain in mental health care emerged from the data analysis; quotations illustrating the motivating factors are in **Table 4** and demotivating factors are in **Table 5**. Motivating factors included 1) a desire to help patients who are vulnerable and in need, 2) positive day-to-day interactions with patients, 3) intellectual or academic interest in psychiatry or behavior, and 4) good relationships with colleagues. Demotivating factors included 1) lack of resources at the hospital, 2) a rigid supervisory hierarchy, 3) lack of positive or negative feedback on work performance, and 4) few opportunities for career advancement within mental health.

**Table 4:** Quotations illustrating motivating factors.

Factor	Quotations
Patient need and vulnerability	“I have a passion to be able to help vulnerable, the vulnerable, and I see psychiatric patients as very vulnerable people.” –Psychiatric nurse
	“This is a group that is marginalized. This is a group that needs the most help. This is a group that don’t have the staff, the personnel to help them.” – Psychiatrist
	It is “the church of nursing, worship in the form of nursing.” – Nurse in leading administrative role
Positive patient interactions	“I mostly like to interview patients, be nice with them, be friends with them.” – Nurse assistant
	“You get to work at least at the end of it you know somebody will create a laughter. Even a patient. Some of them will make you laugh.” – Psychiatric nurse
	“When you look at how neatly they have dressed and they come to you and you talk with them you feel satisfied.” – Psychiatric nurse
	“As a clinician, I feel very fulfilled when a patient or a client was brought in a very bad state, very aggressive, very ill of something. And then after a week or two…you can’t see a difference between the person and a normal, so called normal, person.” – Psychiatrist
Intellectual interest	“To me the job is challenging, and I like challenging jobs. I like, it keeps me thinking, keeps me on my toes, keeps me active.” – Hospital administrator
	“I realized more the interest here is better than the general side because here you really learn how to accommodate human beings.” – Psychiatric nurse
Improved family and interpersonal relationships	“I know much about mental health. I know frustrations that comes to one’s life here and there. And anytime I’m faced with any reality, I’m able to adjust faster because of what I’ve learned in school and what I’ve seen practically.” – Nurse assistant “
	“I tend to understand human nature better, and I think that’s really helping how I can relate to people.” – Psychiatric nurse
	You really learn how to deal with human beings when it comes to problems solving.” – Psychiatric nurse
Relationships with colleagues	“One thing that motivates us to work mostly is our colleagues. Sometimes you come and you are tired, but your colleague says, ‘my friend, let’s get up and do the work.’ and so I am motivated to just go.” – Psychiatric nurse
	“For the counseling unit, we have a family meeting every morning. We pray together, we sing, and we make our target goal then. At the end of the day, this is what we want to achieve.” – Psychiatric nurse

**Table 5:** Quotations illustrating demotivating factors.

Factor	Quotations
Lack of resources: work environment and infrastructure	“If the remuneration is not that remunerating, that’s, that’s not motivating enough. You are not rewarded.” – Psychiatrist
	“You’ve seen it at Accra Psychiatric Hospital. Not too many people are proud of working in such environments.” – Psychiatric nurse
No compensation for high risk environment	“I’m a specialist in psychiatry. A specialist within internal medicine, he’s not exposed to the same dangers I am exposed to. They attack us all the time. For example, these new patients, they attack us several times, they knock at us, break our teeth, but I take the same salary as a specialist in another specialty.” –Psychiatrist
Rigid hierarchy	“Those ahead of you are superiors so they look down on you, they, the manner they might even address you is… very intimidating.” – Psychiatric nurse
	“You can’t help someone who is senior here, even if that person is doing something wrong.” – Psychiatric nurse
Lack of accountability or feedback	“If people don’t see that their work has any value and nobody appreciates them for their work they do, then they obviously will do it anyhow.” – Psychiatrist
	“People want to see an increment in their salaries. People want to see an extra envelope coming from their bosses saying thank you for the extra work that you are doing, but that is not coming.” – Psychiatric nurse
	“Most of our staff, we are very apathetic because that [monetary reward] is not coming from management, for example.” – Psychiatric nurse
	“People doing extra things to help run the hospital are never spotted and you know, say, ‘Oh, that is a well done, thumbs up for you.’ That is not there. So people then get, realize that ‘Oh, if I continue to do this, it does not get recognized, why then do it at all?’” – Psychiatric nurse
Drive to advance	“I’m here because I want to finish my course and be able to do better than work in an institution like this.” – Psychiatrist

Some respondents did not specifically choose to work in mental health, but rather had positions in psychiatric hospitals due to chance or government decisions to address workforce gaps in health services. Even those who did not choose mental health care found aspects of the work that were motivating and rewarding and that kept them working in the psychiatric hospital, despite continued challenges. Following are the key motivating and demotivating factors, illustrated by direct quotations of the key informants in **Tables 4** **and** **5**.

### Motivating Factors

#### Patient need and vulnerability

Nearly all respondents emphasized that they felt drawn to help their patients because of a personal calling to serve those in need. Many saw people with mental health disorders as among society’s most vulnerable because they are stigmatized, hated or ignored by their families, and unable to care for themselves. Staff often linked their drive to help the marginalized to religious faith, with some respondents conceptualizing mental health care as a way of serving God or fulfilling a Christian duty. Workers’ commitment to addressing need extended beyond service to individuals. Respondents described the lack of workers in mental health care and their desire to fill gaps in the health system. They also indicated that they felt galvanized to fight the social stigma surrounding mental health, referencing a desire to correct misunderstandings about mental illness, show others that mental health conditions are treatable, or increase respect for the field of mental health care.

#### Positive patient interactions

Many staff said that they enjoyed their work because of daily contact with inpatients or outpatients. Positive aspects of routine patient care included building close, family-like relationships with patients and aspects of patient behavior, such as stories about life circumstances or wild antics, which staff found interesting or funny.

In addition to discussing routine interactions with patients, staff who regularly worked directly with patients described feeling fulfilled when their patients recovered. Signs of recovery included leaving the hospital, returning to family life, getting a job, and being well dressed. After hospital discharge, most patients did not return to the hospital or went only to the outpatient clinic, not to the ward where they had been treated. As a result, ward staff (primarily nurses), did not see positive treatment outcomes unless their patients returned to the hospital for follow-up appointments and made an effort to come see them on the ward. Nurses noted that they felt satisfied when recovered patients came back to visit the wards, and some nurses went to check-on discharged patients at home, even if it was outside of their professional duties.

#### Intellectual interest

Both staff members with professional training in mental health and those without, including physicians, nurses, administrators, and clinical support staff, cited that they started or continued to work in mental health care because it was more intellectually stimulating than other areas of medicine. When asked about why they work in a psychiatric hospital staff said they found mental health care “interesting” or “adventurous.”

Mental health workers who had post-secondary education in psychiatry or other health sciences, such as physicians, nurses, and pharmacists, saw psychiatry as more challenging than other fields of medicine. Rather than being demotivated by the difficulty of psychiatry, workers noted that the complexity of the field was one of the aspects that drew them to mental health care. Nurses and physicians described the cognitive puzzle of learning about their patients’ interesting lives then assembling the pieces to develop a diagnosis or treatment plan. Staff, particularly those with higher educational backgrounds, emphasized the intellectual challenge of working in mental health, expressing a desire to understand how the human mind works and cited examples of particularly interesting patient cases or scientific mechanisms.

#### Close relationships with colleagues

Staff noted that they continued to work at a psychiatric hospital because of their relationships with their colleagues. Motivating relationships were generally with direct peers, such as others working on the same ward, rather than with people working in other areas of the hospital or supervisors. Nurses often worked on wards with the same team of colleagues for a considerable length of time and discussed the close relationships that developed on the ward. Many nurses said that they enjoyed coming to work because of the relationships they had with the other staff on their ward. According to some nurses, people working in mental health were friendlier than those in general medicine because they had a better understanding of human behavior. Others described how staff on a ward would form close ties, eating, praying, and sharing common struggles at work. Staff also expressed that they felt like part of a team or “family” on a ward, which made them feel invested in the success of the group or drove them to aid colleagues so they could get the same help in return.

#### Improved family and interpersonal relationships

Mental health workers who had less post-secondary education and who and interacted most directly with patients, including nurses and other clinical support staff, reported that they wanted to work in mental health care because it helped them learn how people behave, which improved their daily, non-work interactions and relationships. The skills and understandings that nurses developed at work helped them better understand and navigate complex interpersonal situations. For example, because a nurse had gained a better understanding of others’ motivations through her work, she described feeling better prepared to deal with family conflict. She reported realizing that she should nag her husband less because it drove him to seek other women. Other nurses commented that their experience in mental health care helped them in situations of interpersonal tension outside of work, such as influencing friends and family and interacting with large groups.

### Demotivating Factors

#### Lack of resources: work environment and infrastructure

Respondents highlighted that lack of resources at the psychiatric hospitals made their work more difficult and made mental health care less attractive than other areas of medicine or other professions. The resource limitations were most apparent in staffing, pay, and the hospital environment. Workers were overwhelmed by the enormous patient-to-staff ratio and often had to work long hours, typically without extra pay. Given the heavy patient loads, staff indicated that they were not able to provide what they knew as high quality care for patients and described feeling disappointed because they could not give the level of care that they had been trained to offer.

Additionally, workers reported that they did not earn enough income to allow them to live comfortably or properly educate their children. Some nurses, a position that was in particularly high demand in the private sector, worked second jobs in private hospitals in order to generate additional income. They acknowledged that they were making less money than they could working abroad, in the private sector, or in some areas of general medicine.

Last, the inadequate work environment disheartened staff. They found the dilapidated buildings and poor appearance of the hospital grounds, lack of proper toilets, and limited tools, from basic medical supplies to computers for patient recordkeeping, frustrating. Many respondents noted that they did not feel proud of where they worked because of the poor conditions of the hospital. Workers in a variety of clinical roles also said that they knew how to give higher quality patient care, but their environment prevented them from doing so, leading to frustration and apathy.

#### No compensation for high risk environment

Both physicians and nurses expressed a desire for a “risk allowance” for mental health workers, saying that staff in psychiatric hospitals should earn more money than those in general medicine because they were more likely to be injured by violent patients. Staff described being routinely unable to defend themselves from patient aggression, noting that, in some instances, patients outnumbered staff by a ratio of fifty-to-one and that, without adequate supplies of medication, patients were more likely to become aggressive and threaten staff well being. A number of respondents described injuries that they or their colleagues had incurred as a result of patient attacks and expressed anger and disappointment that there was no easy way to obtain compensation or medical treatment for those injuries. Fear of attacks from patients and frustration about lack of risk compensation led staff to question their commitment to working in mental health care.

#### Rigid hierarchy

Respondents shared a number of incidents in which the hierarchical management system of the hospital inhibited their motivation to work. Workers explained that it was inappropriate to question older or higher-ranking staff members and that those people could be threatening and mean to more junior staff. Respondents who noted this tension expressed resentment about the rigid power structure and said that interpersonal interactions with their superiors made their work environment unpleasant. One staff member shared his desire to leave the hospital because his superiors intimidated him.

#### Lack of accountability or feedback

Respondents complained about the lack of merit-based feedback. Using the words “motivation” and “rewards” synonymously, some workers noted that they sometimes received “non-tangible motivation,” verbal praises for their work, but explained that verbal feedback was not enough to motivate them in the long-term; they wanted to work toward an increase in pay or responsibility. Nurses, clinical support staff, and administrators were automatically promoted every five years, regardless of the quality of their work. This reduced their incentive to work hard because effort and initiative were not linked to career advancement. Furthermore, administrators said that the culture of the hospitals dictated against firing staff members under any circumstances, which gave staff leeway to underperform. Staff also indicated that even non-tangible rewards were missing, explaining that managers did not verbally acknowledge staff who worked unpaid overtime, spent their own money to buy supplies for the hospital, or achieved good patient outcomes. Although two participants had received awards from the hospital or nurses’ association for their high quality work, both the award winners and other respondents acknowledged that awards were rare and given only sporadically, and there was limited guidance on how to work toward them or toward opportunities for promotion or career advancement.

Many workers describe the effect that the paucity of feedback had on their colleagues: “most of our staff, we are very apathetic,” one nurse manager explained, citing lack of recognition or reward from management as the main cause of apathy. The lack of feedback left staff feeling unappreciated, which respondents specifically linked to poor performance: workers sitting idly, not interacting with patients, and being habitual tardy.

#### Few opportunities for career advancement in mental health

Respondents in both clinical care and administrative support roles expressed a desire to leave the field of mental health in order to further their careers. Working in a psychiatric hospital was seen as a stepping stone to careers outside of public mental health care, such as a job in private medical care, human resource management, or business. Staff explained that it was not difficult to get a job in a psychiatric hospital, and that position provided preparatory experience for other jobs. A number of respondents said that they hoped to leave the psychiatric hospital as soon as possible and were just waiting for positions abroad or in other areas to become available or feasible.

Additionally, even if they were interested in a career in mental health care, staff members who wanted more academic opportunities or more education felt pressure to leave the field. Physicians noted that they could not do research or pursue their academic interests because the patient burden was greater than in other areas of health care. Many nurses wanted to get additional training or degrees in order to further their careers, but while additional nursing training was available in general medicine and midwifery, no such training in psychiatry was offered in Ghana. Nurses said they would have to move to another field of health care if they wanted to get the advanced training that would lead to higher paying positions.

## Discussion

The data suggest that the motivation of hospital workers to remain in mental health care in a low-income country may be particularly influenced by interpersonal interactions and group dynamics. A common theme running through the motivating and demotivating factors is the quality of interactions between workers and their peers, supervisors, patients, friends, or family members. Staff were drawn to mental health care because of close, family-like relationships with colleagues or patients; constructive and regular feedback from supervisors; amusing, rewarding, or edifying contact with patients; and learning how to improve their relationships with family and friends. They were demotivated by supervisors who were overly harsh or dominant and by lack of personal recognition for their work. As efforts are made to improve working conditions in order to retain those in mental health care, the role of interpersonal interactions must be carefully considered. It is a central factor in workers’ satisfaction and may cost less to address than increasing compensation, improving hospital appearance and infrastructure, or expanding training programs, other key motivating factors. Although stronger relationships may primarily improve conditions for existing workers, our results also highlight the importance of infrastructure investments and elements of stigma reduction, which may affect recruitment, helping draw new workers into the field.

No previous studies have examined factors that motivate mental health workers or have highlighted the role that relationships play in retention of this population of health workers. Although our study is consistent with previous research on health worker retention in low-resource settings, which explains that low pay, a poor work environment, and inadequate supervision reduce retention [7], [8], [9], [10], [11], our findings also reveal that interpersonal interactions are critical to the motivation of mental health workers.

Rather than focusing on dynamics between individuals or within groups, much of the previous literature has centered on factors that may be expensive to address and, as a result, difficult to tackle on a broad scale, such as increasing salaries, expanding educational programs, and improving the hospital infrastructure. Relationships, however, could be enhanced by strengthening hospital leadership and management, a change that does not necessitate substantial output of resources. Previous studies on hospital leadership and management have shown that interventions to develop these features are feasible and effective in low-income countries [29], [30], [31], [32]. Additional research is needed, however, to know how useful these interventions might be in mental health.

Interventions to improve leadership and management in psychiatric hospitals could enhance factors that motivate staff, while mitigating factors that we found to be demotivating. For example, efforts aimed at developing a more adaptable and open organizational hierarchy could facilitate communication among staff of different ranks, allow merit-based advancement, and enable supportive and open relationships with supervisors. Additionally, creating specific metrics to assess worker performance could enhance transparent accountability and increase the quantity and quality of feedback given to staff, which, in turn, could lead to increased motivation. Finally, team-building efforts could strengthen interpersonal relations throughout the hospital and increase commitment to the institution.

In addition to highlighting potentially low-cost ways to improve retention of mental health workers, this study underscores key areas where resources could be invested to improve working conditions, aiding in both recruitment and retention. Establishing risk pay for psychiatric workers may motivate the current staff, while also attracting more health workers to the field, rather than choosing to work in other areas of health care. Rewarding high quality work with extra pay or responsibility, providing adequate tools for patient care, and improving the appearance of hospitals may make workers feel more successful, motivated, and connected with their patients.

Our results also highlight the stigma that surrounds mental health in Ghana. This stigma affects not only people with mental health disorders and their families, but also the people who build careers caring for them, an element of stigma not highlighted by previous research in low and middle-income countries. In addition to noting that their patients were stigmatized, respondents noted that they had fewer career development and educational opportunities than staff in other areas of medicine. A number of staff asserted that they enjoyed mental health care because they found the field more intellectually engaging than other areas of medicine, suggesting that educational opportunities may be particularly important for health workers in this field. Additionally, some respondents noted that they may not have initially wanted to enter mental health care and had known little about it. It was only after they overcame the social stigma by spending time with patients with mental health disorders, learning more about mental illness, and getting to know their colleagues that they came to enjoy and appreciate their work.

Because of the pervasive stigma, interventions that foster greater understanding of mental health disorders and treatments, both among the general public and policymakers, are paramount. Such interventions may help retain current workers by facilitating the creation of professional development and educational opportunities, ensuring that there is an advancement track comparable to those in other areas of medicine. They may also bring new workers into the field by dispelling misunderstandings about mental illness and helping people understand that mental illnesses are treatable, like physical health conditions.

Our findings should be understood in light of several limitations. The study was conducted in Ghana, and the themes may differ in other low and middle-income countries. Given the paucity of data on the experiences of mental health workers, however, it is valuable to gain an in-depth understanding of a certain area before conducting a multi-country study. Additionally, this was an exploratory, qualitative study, designed to generate ideas and hypotheses about factors that motivate or demotivate mental health workers. Larger, quantitative studies are needed to determine the statistical associations between motivating or demotivating factors and successful recruitment and retention of mental health workers; yet because little research had been conducted on the experiences of mental health workers in low- and middle-income countries, a qualitative study was appropriate. There is also a risk that the validity of the data was compromised because respondents did not answer the questions openly and honestly. In order to reduce this risk, we used a number of tactics to promote candor in the interviews, including allowing respondents to choose not to be recorded and using an open-ended interview guide. The length of the code reports and the richness of personal experiences in them suggest that there was considerable depth in the interviews. Finally, we conducted interviews with mental health workers in institutional settings only. Although these workers may have different motivating factors than community mental health workers, the majority of mental health workers in Ghana work in large hospitals, making a detailed understanding of this segment of the workforce a priority. Future studies should aim to identify if differences in motivation for community-based mental health workers exist.

Ghana is a particularly important country in which to research mental health systems because it is at the forefront of mental health policy development among African nations. In March 2012, Ghana’s Parliament passed legislation to overhaul the mental health system. The bill codified broad reforms, including efforts to strengthen and expand the workforce [33]. The depth of and human rights provisions in the bill differentiate the strength of Ghana’s new mental health law from many other low-income countries, making it a model for other nations. The WHO was involved in the drafting of the bill, and its passage builds on a resolution from the Executive Board of the WHO’s World Health Assembly urging member countries to increase resources for and attention to mental health [34]. The new international focus on mental health system strengthening and the policy reforms within Ghana suggest that now is a key time to develop effective interventions for improving mental health service delivery.

Our analysis has revealed the importance of interpersonal interactions and group dynamics in mental health worker motivation. Qualitative methods allowed us to gain an in-depth understanding of the experiences of mental health workers and to expose ideas for recruitment and retention that may have otherwise gone unrecognized. Our findings suggest a set of resource-efficient approaches to bolstering the mental health workforce: training for hospital staff in leadership and management, investment in key infrastructure improvements, and a focus on reducing the pervasive stigma surrounding mental health. These findings can be leveraged to expand access to mental health care by informing the development of evidence-based and low cost strategies to increase recruitment and retention of mental health workers.
